# Design Strategies of PEDOT:PSS-Based Conductive Hydrogels and Their Applications in Health Monitoring

**DOI:** 10.3390/polym17091192

**Published:** 2025-04-27

**Authors:** Yingchun Li, Xuesi Zhang, Shaozhe Tan, Zhenyu Li, Jiachun Sun, Yufeng Li, Zhengwei Xie, Zijin Li, Fei Han, Yannan Liu

**Affiliations:** 1 Advanced Interdisciplinary Research Center for Flexible Electronics, Academy of Advanced Interdisciplinary Research, Xidian University, Xi’an 710071, China; 2 State Key Laboratory of Wide-Bandgap Semiconductor Devices and Integrated Technology, Faculty of Integrated Circuit, Xidian University, Xi’an 710071, China; 3 Shaanxi Key Laboratory of Degradable Biomedical Materials, School of Chemical Engineering, Northwest University, Xi’an 710069, China; 4 The Key Laboratory of Biomedical Information Engineering of Ministry of Education, School of Life Science and Technology, Xi’an Jiaotong University, Xi’an 710049, China; 5 Bioinspired Engineering and Biomechanics Center (BEBC), Xi’an Jiaotong University, Xi’an 710049, China

**Keywords:** hydrogel, sensor, conductive, PEDOT:PSS, flexible electronic

## Abstract

Conductive hydrogels, particularly those incorporating poly(3,4-ethylenedioxythiophene):polystyrene sulfonate (PEDOT:PSS), have revolutionized wearable health monitoring by merging tissue-like softness with robust electronic functionality. This review systematically explores design strategies for PEDOT:PSS-based hydrogels, focusing on advanced gelation methods, including polymer crosslinking, ionic interactions, and light-induced polymerization, to engineer hierarchical networks that balance conductivity and mechanical adaptability. Cutting-edge fabrication techniques such as electrochemical patterning, additive manufacturing, and laser-assisted processing further enable precise microstructural control, enhancing interfacial compatibility with biological systems. The applications of these hydrogels in wearable sensors are highlighted through their capabilities in real-time mechanical deformation tracking, dynamic tissue microenvironment analysis, and high-resolution electrophysiological signal acquisition. Environmental stability and long-term durability are critical for ensuring reliable operation under physiological conditions and mitigating performance degradation caused by fatigue, oxidation, or biofouling. By addressing critical challenges in environmental stability and long-term durability, PEDOT:PSS hydrogels demonstrate transformative potential for personalized healthcare, where their unique combination of softness, biocompatibility, and tunable electro-mechanical properties enables seamless integration with human tissues for continuous, patient-specific physiological monitoring. These systems offer scalable solutions for multi-modal diagnostics, empowering tailored therapeutic interventions and chronic disease management. The review concludes with insights into future directions, emphasizing the integration of intelligent responsiveness and energy autonomy to advance next-generation bioelectronic interfaces.

## 1. Introduction

Conductive hydrogels have emerged as promising materials for next-generation wearable sensors, owing to their unique blend of electrical conductivity, mechanical flexibility, and biocompatibility [1,2,3,4]. These materials combine the high water content and inherent softness of conventional hydrogels with electronic functionality, making them ideal for flexible electronics, bioelectronics, and health monitoring applications [5,6,7,8]. Their ability to maintain conductivity while retaining moisture facilitates an effective interface with biological tissues, ensuring both comfort and optimal performance in wearable sensing platforms [9,10]. The tunable mechanical properties of these hydrogels—achieved via adjustments in crosslinking density, polymer concentration, and network architecture—allow their elastic modulus to be tailored within the range of 1–100 kPa, closely matching the compliance of human soft tissues (e.g., skin: ~10–100 kPa; muscle: ~8–15 kPa) [11]. This adaptability ensures smooth conformity to dynamic body movements (e.g., joint flexion, muscle contraction) by minimizing interfacial stress concentrations and maintaining mechanical integrity during cyclic deformation. Such characteristics render them well suited for real-time physiological monitoring and long-term biomedical applications [12,13,14,15].

Among the wide array of conductive hydrogels, those based on poly(3,4-ethylenedioxythiophene):polystyrene sulfonate (PEDOT:PSS) have attracted considerable attention due to their superior electrical performance, environmental stability, and versatile processability [16,17]. PEDOT:PSS hydrogels can be constructed using a variety of gelation methods, as outlined in recent research: polymer crosslinking techniques, which include physical, chemical, and combined physical–chemical approaches, offer a controllable means to achieve the desired network structure [18,19,20]; ionically induced gelation leverages ionic interactions to form stable gels [21,22]; and photo-induced gelation provides spatial and temporal control over the crosslinking process [23]. Additionally, advanced fabrication schemes such as casting and molding, wet spinning, electrospinning, electrochemistry, inkjet printing, direct ink writing, and digital light processing (laser-induced) have further broadened the design space, enabling precise control over the hydrogel’s microstructure and performance characteristics [24,25,26,27,28].

**Figure 1:**
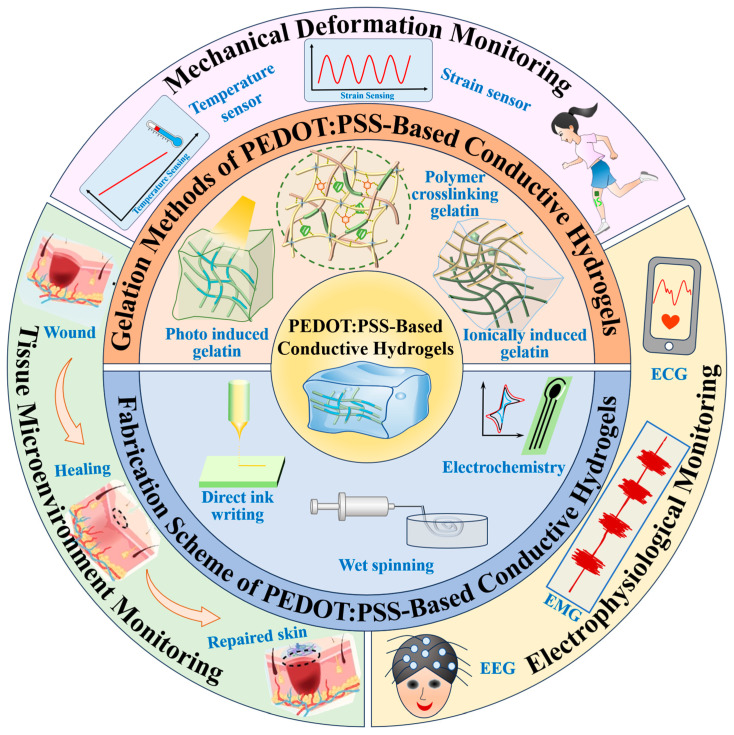
Overview of construction strategies and emerging applications of PEDOT:PSS-based conductive hydrogels in health monitoring.

This review provides a comprehensive overview of the design strategies and fabrication techniques for PEDOT:PSS-based conductive hydrogels, and explores their diverse applications in health monitoring (Figure 1). The discussion is organized into key sections covering the construction strategies, including the various gelation methods and fabrication schemes, and the practical applications in wearable sensors for monitoring mechanical deformations, tissue microenvironments, and electrophysiological signals. By summarizing recent advances and outlining future perspectives, this work aims to address critical challenges and guide the development of next-generation conductive hydrogel-based technologies for personalized and intelligent healthcare solutions.

## 2. Construction Strategies of PEDOT:PSS-Based Conductive Hydrogels

### 2.1. Gelation Methods of PEDOT:PSS-Based Conductive Hydrogels

Conductive hydrogels based on PEDOT derivatives (PEDOTs) have emerged as promising materials for applications in flexible electronics, biosensors, and tissue engineering due to their unique combination of electrical conductivity, mechanical flexibility, and biocompatibility [5,8]. The construction of these hydrogels relies on rational design strategies to integrate conductive PEDOTs into hydrogel networks while maintaining structural integrity and functionality [10]. Gelation is a pivotal step in forming stable hydrogel networks. For PEDOT-based hydrogels, the gelation process must balance the incorporation of conductive polymers with the formation of a crosslinked hydrophilic matrix. Gelation is a pivotal step in forming stable hydrogel networks. For PEDOT-based hydrogels, stability requirements include (1) sufficient mechanical resilience to withstand physiological stresses (e.g., cyclic stretching, compression) without fracture or plastic deformation; (2) resistance to swelling/deswelling-induced structural collapse under varying humidity or temperature; (3) long-term retention of electrical and mechanical properties to prevent performance degradation caused by oxidation, hydrolysis, or biofouling; and (4) biocompatibility, ensuring no cytotoxic byproducts or leachable crosslinkers remain post-gelation [8,10]. Achieving these criteria necessitates balancing the incorporation of conductive polymers with the formation of a crosslinked hydrophilic matrix through tailored gelation strategies. The relative advantages and disadvantages of physical, chemical, and hybrid crosslinking methods are summarized in Table 1.

#### 2.1.1. Polymer Crosslink Gelatin

**Figure 2:**
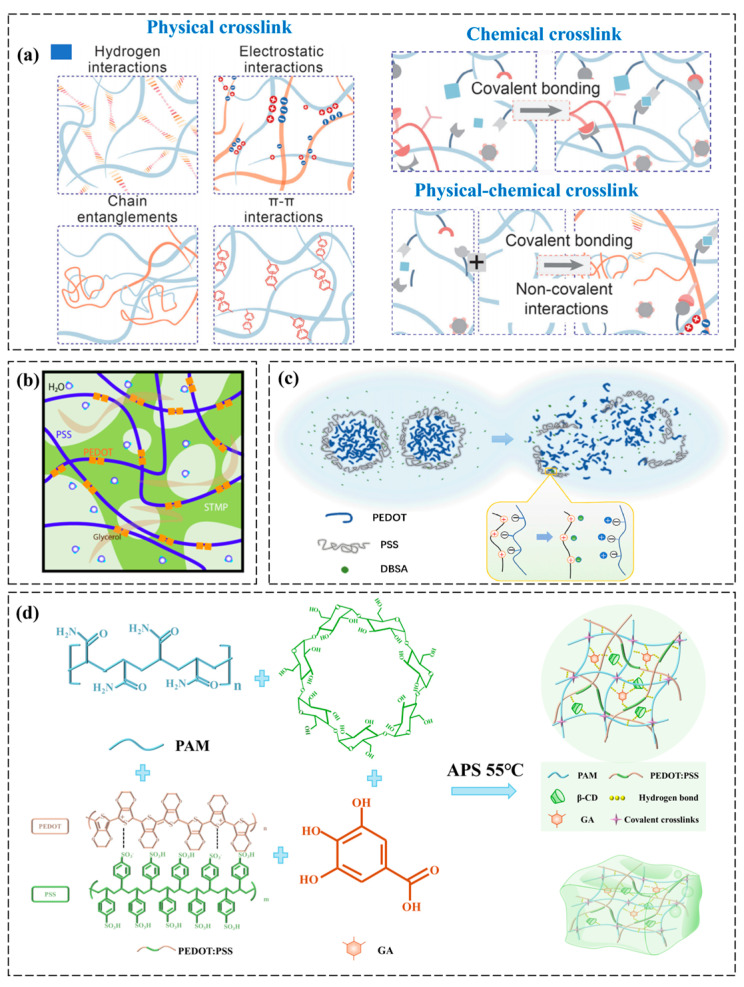
Gelation methods of PEDOT:PSS conductive hydrogels based on polymer crosslinking. (**a**) Schematic illustration of three distinct crosslinking mechanisms: physical crosslinking mediated by non-covalent interactions, including hydrogen bonding, electrostatic attraction, polymer chain entanglements, and π-π stacking; chemical crosslinking achieved through irreversible covalent bond formation; hybrid physico-chemical crosslinking combining both covalent bonding and dynamic non-covalent interactions [29]. (**b**) Schematic illustration of the interpenetrating PEDOT:PSS conductive hydrogel network, where sodium trimetaphosphate (STMP) acts as a dynamic ionic crosslinker to facilitate unrestricted ion mobility within the 3D polymeric matrix [30]. (**c**) Schematic illustration of DBSA-mediated surface restructuring in PEDOT:PSS particles through surfactant-assisted PSS stripping, revealing enhanced charge transport pathways [32]. (**d**) Schematic diagram of the synthesis steps and synthesis conditions of conductive hydrogels through thermal copolymerization method involving radical grafting and supramolecular self-crosslinking reactions [33]. All pictures have adopted with permission.

Polymer crosslinking strategies are widely employed to create robust hydrogel networks, including physical crosslinking, chemical crosslinking and physical–chemical crosslinking (Figure 2a) [29]. Physical crosslinking is achieved through the utilization of non-covalent interactions such as hydrogen bonding, hydrophobic interactions, π-π stacking, and electrostatic forces. For example, Reynolds et al. engineered an interpenetrating network of conductive polymer hydrogels with programmable electrical and mechanical characteristics. By leveraging sodium trimetaphosphate-mediated dynamic physical crosslinking with PEDOT:PSS, they achieved a glycerol-free hydrogel system exhibiting enhanced conductivity, tunable wettability, and robust shear-thinning rheology (Figure 2b) [30]. However, physically crosslinked PEDOT:PSS hydrogels typically exhibit elastic moduli in the range of 1–10 kPa, which is significantly lower than chemically crosslinked systems (e.g., 10–100 kPa) due to the reversible nature of non-covalent bonds [30]. In addition, PEDOT:PSS can form physically crosslinked networks with natural polymers (e.g., gelatin, chitosan) through hydrogen bonding [31]. While this method avoids toxic crosslinkers, the resulting hydrogels may exhibit limited long-term stability in aqueous environments due to reversible bond dissociation, further restricting their mechanical performance under cyclic strain.

Chemical crosslinking is achieved by introducing covalent bonds through crosslinking agents (e.g., glutaraldehyde, genipin) or photoinitiators, enabling robust and stable network formation in polymer matrices. Liu et al. fabricated a double-crosslinked hydrogel via in situ chemical crosslinking within a poly(vinyl alcohol) (PVA) matrix. Dodecyl benzene-sulfonic acid (DBSA) not only partially removed surface PSS from PEDOT:PSS but also induced a uniformly distributed porous architecture (PVA-PP-DBSA), achieving a sixfold enhancement in tensile strain (90% to 580%) while establishing an interconnected conductive network (Figure 2c) [32]. PEDOT:PSS can be grafted onto acrylamide-based hydrogels via free-radical polymerization, enhancing both conductivity and mechanical strength. However, residual crosslinkers may raise biocompatibility concerns.

Physical–chemical crosslinking employs hybrid strategies that synergistically integrate covalent bonds (via chemical crosslinking agents) and reversible non-covalent interactions (e.g., hydrogen bonds or π-π stacking), enabling tunable mechanical robustness and dynamic adaptability in polymer networks. For instance, Li et al. developed a multifunctional hydrogel sensor via thermal copolymerization combining radical grafting (chemical crosslinking) and supramolecular self-crosslinking (physical interactions). The hydrogel fabrication involved sequential dissolution of acrylamide (AAM), β-CD, N,N’-methylene bis-acrylamide, and GA in a glycerol–water solution, followed by PEDOT:PSS dispersion and ammonium persulfate-initiated thermal polymerization to form the dual-crosslinked network (Figure 2d) [33]. This hybrid approach synergistically enhanced elasticity and fatigue resistance compared to single-method gelation: covalent bonds provided structural integrity (elastic modulus ~25 kPa), while supramolecular interactions (e.g., hydrogen bonds between β-CD and PEDOT:PSS) enabled dynamic energy dissipation during cyclic loading. As a result, the dual-network hydrogel achieved a tensile strain of ~800%—significantly exceeding the performance of purely chemically crosslinked analogs (~300–500%)—and retained 90% of its initial conductivity after 1000 stretching cycles at 50% strain. In contrast, hydrogels relying solely on physical crosslinking exhibited irreversible network breakdown under similar conditions, while chemically crosslinked versions suffered from brittle fracture due to limited energy dissipation mechanisms.

#### 2.1.2. Ionically Induced Gelatin

**Figure 3:**
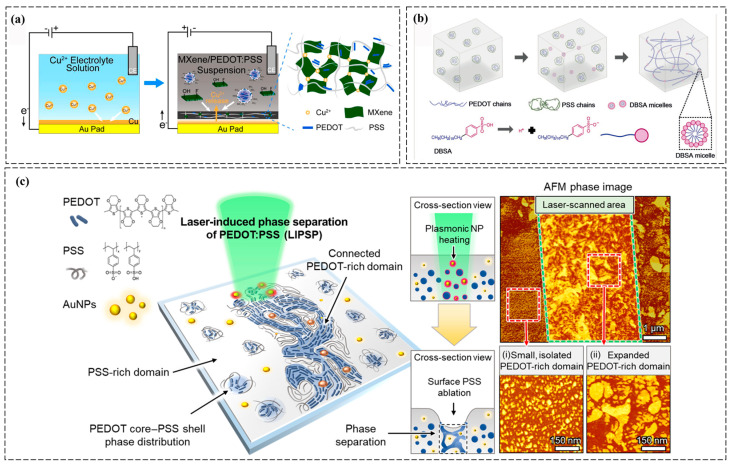
Gelation methods of PEDOT:PSS-based conductive hydrogels. (**a**) Schematic illustration of porous MXene/PEDOT:PSS suspension hydrogel fabrication using copper as a sacrificial metal and its resulting three-dimensional network structure [20]. (**b**) The crosslinking mechanism of PEDOT:PSS hydrogel involves DBSA-induced weakening of electrostatic interactions between PEDOT⁺ and PSS⁻, exposing PEDOT⁺ chains to water, which undergo a coil-to-linear conformational change and physically crosslink via π–π stacking and hydrophobic interactions [37]. (**c**) The laser-induced phase separation of PEDOT:PSS process uses laser-induced photothermal effects on AuNPs to ablate surface PSS and induce phase separation, with AFM images revealing PEDOT-rich and PSS-rich domains in the laser-scanned and nontreated regions [38]. All pictures have adopted with permission.

Ionic crosslinking exploits multivalent ions (e.g., Ca^2+^, Fe^3+^) to bridge polymer chains. For example, alginate-PEDOT:PSS hydrogels can be formed by immersing alginate solutions in Ca^2+^ baths, where Ca^2+^ ions crosslink alginate chains while PEDOT:PSS enhances conductivity. While this method is simple and scalable, long-term stability in ionic environments (e.g., physiological fluids or sweat) is often compromised due to (1) competitive ion exchange (e.g., displacement of Ca^2+^ by Na^+^ or K^+^ in biological fluids), which weakens crosslinking density; (2) the reversible nature of ionic bonds, leading to gradual dissociation under sustained mechanical stress; and (3) swelling-induced structural disintegration in high-ionic-strength solutions, as osmotic pressure disrupts the hydrogel network [34]. Zhang et al. developed a 3D porous Ti_3_C_2_T_x_ MXene/PEDOT:PSS composite aerogel (MPCA) via Cu-assisted electrogelation with controllable patterning. The strong MXene–PEDOT:PSS interactions form a stable 3D conductive network, enhancing mechanical flexibility and piezoresistive performance. The resulting pressure sensor exhibits high sensitivity (26.65 kPa^−1^, 0–2 kPa), fast response (106 ms), and excellent stability for wearable monitoring. Leveraging its patternability, a high-resolution sensor array was integrated into a robotic fingertip for tactile recognition, including braille detection, highlighting MPCA’s potential in next-generation wearable electronics (Figure 3a) [20]. Moreover, a 4 wt% PEDOT:PSS hydrogel with ultra-high conductivity (880 S m^−1^) was fabricated via thermal and acid treatment, and shaped into various forms. A porous fiber enabled a flexible, collector-free supercapacitor with high capacitance (202 F cm^−3^), excellent rate performance, and strong potential for wearable energy storage [21].

Ionic liquids (ILs) serve as green solvents or crosslinkers due to their high ionic conductivity and non-volatility. ILs like 1-ethyl-3-methylimidazolium tetrafluoroborate ([EMIM][BF_4_]) can dissolve PEDOTs and facilitate their dispersion in hydrogel precursors [35]. The resulting hydrogels exhibit enhanced conductivity and thermal stability, making them suitable for high-performance wearable devices [36]. For example, Zhang et al. developed injectable PEDOT:PSS/4-dodecylbenzenesulfonic acid (DBSA) hydrogels that spontaneously form at room temperature without additional treatment. A simple method also enables the large-scale production of hydrogel fibers. These RT-formed hydrogels and fibers are promising for soft, self-healing bioelectronic devices (Figure 3b) [37]. Liu et al. developed soft, conductive hydrogel-based “elastronic” microelectronics with kilopascal-range modulus, integrating a high-capacitance hydrogel conductor and elastic fluorinated photoresist insulation. The 20 μm electrode arrays exhibit ~30× higher current-injection density than platinum, low interfacial impedance, and stable performance under strain, enabling localized low-voltage sciatic nerve stimulation in vivo [22].

#### 2.1.3. Photo-Induced Gelatin

Photoinitiated gelation enables spatiotemporal control over hydrogel formation. In methacrylated polymers (e.g., gelatin methacryloyl, GelMA), UV or visible light (typically 365–405 nm wavelength) activates photoinitiators (e.g., Irgacure 2959) to generate free radicals via photolysis. These radicals initiate chain-growth polymerization by attacking the vinyl groups (C=C bonds) in the methacrylate side chains of GelMA, propagating covalent crosslinks between polymer chains to form a 3D network. When blended with PEDOTs, the conductive polymers are entrapped within the hydrogel matrix during photopolymerization. Notably, PEDOT:PSS can interact with methacrylated polymers through hydrogen bonding or π-π stacking, which stabilizes the dispersion of conductive phases while minimally interfering with the radical-mediated crosslinking process [23]. Light-induced methods are ideal for patterning microstructures or fabricating devices with high spatial resolution. For instance, Won et al. developed an ultrafast, biocompatible digital patterning process for PEDOT:PSS hydrogels using laser-induced phase separation. This method enhanced the electrical properties and aqueous stability of PEDOT:PSS, transforming it into water-stable hydrogels. The hydrogels exhibited high electrical conductivity of 670 S/cm with 6 μm resolution in water, maintaining electrochemical stability even after 6 months in a physiological environment. Additionally, stable neural signal recording and stimulation were demonstrated using laser-fabricated hydrogel electrodes (Figure 3c) [38].

### 2.2. Fabrication Technologies of PEDOT:PSS-Based Conductive Hydrogels

#### 2.2.1. Casting and Molding

**Figure 4:**
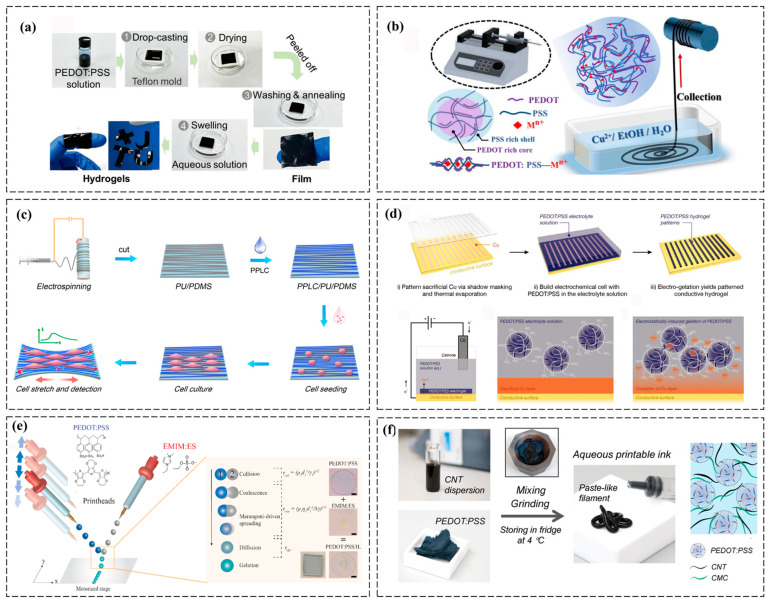
Fabrication technologies of PEDOT:PSS-based conductive hydrogels in health monitoring. (**a**) Preparation process of PEDOT:PSS-based metal halide hydrogels using DMSO as an additive [18]. (**b**) The fabrication process of PEDOT:PSS-Cu^2+^ fibers [39]. (**c**) Schematic illustration of the fabrication process of PPLC/PU/PDMS electrodes and their application in monitoring H_2_O_2_ release from aligned A7r5 cells [42]. (**d**) Patterned electrogels are formed via copper oxidation in a PEDOT:PSS electrolyte, where Cu^2+^-induced microgel assembly replaces the original copper pattern with a hydrogel network [44]. (**e**) Fabrication schematic of patterned PEDOT:PSS/IL and coalescence-driven gelation mechanism with supporting microscopy images [47]. (**f**) Preparation process of the PEDOT:PSS composite inks for DIW [50]. All pictures have adopted with permission.

Casting involves pouring a precursor solution into molds to form bulk hydrogels. This low-cost method is suitable for large-scale production but lacks precision for complex geometries. Molds with micro-/nano-patterns can imprint tailored surface textures to achieve advanced interfacial properties in biosensors, including (1) enhanced bio-tissue adhesion via hierarchical topographies mimicking natural extracellular matrices; (2) increased effective sensing area for improved signal-to-noise ratio in physiological detection; (3) directional strain distribution to minimize interfacial delamination during dynamic motion; and (4) localized control of electrical/ionic pathways for multiplexed signal acquisition (e.g., simultaneous pH, glucose, and mechanical strain monitoring) [18]. For instance, Wang et al. developed metal-halide-doped PEDOT:PSS hydrogels via casting, achieving a record conductivity of 547 S cm^−1^, which is 1.5 times to 10^4^ times higher than filler-free polymeric hydrogels. Ion exchange-induced phase separation forms an ultraconductive network. The hydrogels demonstrate multifunctionality, including thermoelectric generation (1.94 μW m^−1^ K^−2^), EMI shielding (62 dB), Joule heating (62 °C at 1.5 V), and strain sensing (gauge factor 5.3), alongside excellent flexibility (>40% strain) and mechanical stability, making them promising for flexible bioelectronics and wearables (Figure 4a) [18]. However, uncontrolled phase separation can compromise mechanical integrity. Mitigation strategies include additive engineering (incorporating amphiphilic surfactant or ionic liquids to improve interfacial compatibility), solvent optimization (using co-solvents to homogenize polymer blends before curing) and in situ polymerization (direct synthesis of PEDOT within preformed hydrogels avoids pre-mixing challenges) [18].

#### 2.2.2. Wet Spinning

Wet spinning extrudes polymer solutions into coagulation baths to form fibers. PEDOT:PSS/alginate fibers, for example, are spun into Ca^2+^ baths, yielding flexible, conductive threads for textile-based electronics [29]. Challenges include optimizing extrusion rates and bath compositions to prevent fiber brittleness. For instance, inspired by spider fluff, Wang et al. developed an ion-induced self-assembly strategy for the continuous fabrication of PEDOT:PSS fibers with ordered arrays (Figure 4b) [39]. Copper-complexed fluff-like nanostructures spontaneously form on fibers without post-treatment, outperforming conventional methods. The biomimetic fibers exhibit a 5× higher surface area than pristine counterparts, achieving exceptional pressure sensitivity (82 Pa detection limit, 47 ms response). Demonstrated applications include airflow monitoring, real-time signal transmission, and multimodal force sensing via textile integration. This work elucidates microstructure formation mechanisms, revolutionizing wet-spinning technologies and establishing a biomimetic paradigm for high-sensitivity functional fiber design. Furthermore, Sun et al. engineered helical all-polymer microfibers (PU@PVA-PEDOT:PSS) via microfluidic spinning, integrating a strain-insensitive semi-interpenetrating hydrogel core. Optimized processing achieves 500% stretchability, 147 S cm^−1^ conductivity, strain-resilient conductance (ΔR < 5% at 100% strain), and 2000-cycle durability. The helical architecture enables dual-mode sensing (0.15 kPa^−1^ sensitivity) and low-voltage heating (45 °C@1.5 V), demonstrating viability as smart wearables and soft robotic skins. This methodology redefines conductive fiber design principles, bridging mechanical compliance with electronic stability for next-gen flexible systems [40].

#### 2.2.3. Electrospinning

Electrospinning produces nanofibrous hydrogels by applying high voltage to polymer solutions. PEDOTs blended with PVA or poly(ethylene oxide) (PEO) form ultrafine fibers with high surface-area-to-volume ratios, ideal for neural tissue scaffolds [41]. However, achieving uniform dispersion of conductive polymers in nanofibers remains technically demanding [29]. For instance, Bi et al. developed electrospun PU nanofiber-PDMS biointerfaces with PEDOT:PSS-LiTFSI-CoPc coatings, achieving dual bioelectronic functionality. Aligned nanofibers (85% orientation efficiency) guide contractile smooth muscle cell organization while the conductive nanocomposite enables strain-resistant H_2_O_2_ monitoring (3.2% signal drift at 30% stretch, 0.32 μA μM^−1^ cm^−2^ sensitivity). The platform detects hypertension-linked H_2_O_2_ surges (184% baseline elevation) and resveratrol-mediated suppression (62% reduction), providing mechanochemical insights into vascular pathogenesis. This biofabrication strategy bridges electrophysiological sensing with tissue remodeling studies, advancing drug evaluation platforms for cardiovascular diseases (Figure 4c) [42].

#### 2.2.4. Electrochemistry

Electrochemical methods use applied voltages to induce gelation via redox reactions. For example, PEDOT:PSS hydrogels can be synthesized by electrodeposition on electrodes, enabling precise thickness control. This approach is promising for on-chip biosensors but requires conductive substrates [43]. A novel electrochemical method is presented for rapidly patterning PEDOT:PSS hydrogels onto various conductive templates, including curved and 3D surfaces. High spatial resolution is achieved via a sacrificial metal layer that defines the hydrogel pattern, enabling the integration of high-performance conducting hydrogels and aerogels with desirable material properties into increasingly complex device architectures (Figure 4d) [44]. Furthermore, Feng et al. presented a rotational electrospinning strategy combining EDOT-loaded nanofibers with in situ FeCl_3_-mediated polymerization to fabricate aligned, core–shell conductive fibers (σ = 38 S cm^−1^). Continuous FeCl_3_ infusion ensures simultaneous PEDOT sheath formation and alignment, while chloroform etching yields hollow PEDOT microtubes with preserved structure. This dynamic templating and interfacial polymerization approach enables the scalable fabrication of orientation-defined 1D architectures for flexible bioelectronics and energy applications [45].

#### 2.2.5. Inkjet Printing

Inkjet printing deposits hydrogel precursors in precise patterns layer by layer [46]. PEDOT:PSS-based bioinks are tailored for printability (e.g., viscosity, surface tension) to create microelectrode arrays or flexible circuits. Post-printing crosslinking is typically achieved via one or more of the following methods: (1) UV curing: Printed structures are exposed to UV light (e.g., 365 nm) to activate photoinitiators (e.g., Irgacure 2959) within the bioink, generating free radicals that induce covalent crosslinking of methacrylated or acrylated polymers (e.g., GelMA, PEGDA). (2) Ionic crosslinking: Printed layers are immersed in multivalent ion baths (e.g., Ca^2+^ for alginate-based hydrogels) to form reversible ionic bonds. (3) Thermal crosslinking: Heating initiates reactions between temperature-sensitive crosslinkers (e.g., genipin) or accelerates gelation kinetics in pre-mixed systems. (4) Chemical crosslinking: Spraying or vapor-phase exposure to crosslinking agents (e.g., glutaraldehyde) creates covalent networks post-deposition. These processes ensure structural stability and mechanical integrity while preserving patterned geometries [43]. For instance, Bihar et al. developed inkjet-printed conducting polymer electrodes on paper for electrocardiography, enabling high-quality signal acquisition by simply placing two fingers on the device. The recordings remained stable over three months, demonstrating a low-cost, metal-free approach for fabricating medical electrodes with minimal processing, offering promising tools for cardiovascular disease prevention [46]. Moreover, Teo et al. developed microreactive inkjet printing (MRIJP) for programmable 2D/3D patterning of PEDOT:PSS/ionic liquid hydrogels via in-flight reactive merging. The method achieves sub-300 μm droplets (260 ± 15 μm) within 600 μs, leveraging Marangoni-driven self-encapsulation. MRIJP enables mold-free, freeform architectures with electrochemical performance (12.8 mS cm^−1^) comparable to spin-coated films, eliminating post-processing. Demonstrated structures, such as hemispherical biointerfaces and high-aspect-ratio fractal electrodes, highlight its potential for topologically optimized neural interfaces and biosensors with spatially encoded conductivity gradients (Figure 4e) [47].

Certain progress has been made in inkjet-printed PEDOT:PSS circuits. However, residual solvents (e.g., water, DMSO) in casted or printed hydrogels can plasticize the matrix, reducing mechanical stability or causing cytotoxicity [46]. Strategies to address this include post-treatment protocols (freeze–thaw cycles or supercritical CO_2_ drying to remove trapped solvents), crosslinking optimization (UV or thermal curing with multi-functional crosslinkers to stabilize networks while volatilizing solvents) and alternative formulations (solvent-free systems eliminate solvent retention entirely) [43].

#### 2.2.6. Direct Ink Writing

Direct ink writing (DIW), a 3D printing technique, extrudes shear-thinning hydrogels through nozzles to build 3D structures. PEDOT-loaded hydrogels with rheological modifiers (e.g., nanoclay) enable the fabrication of intricate architectures for implantable devices. Challenges include maintaining conductivity during layer-by-layer assembly [48]. A 3D-printable PEDOT:PSS-based hydrogel is developed, offering excellent printability for direct ink writing, tissue-like compliance, strong bioadhesion (interfacial toughness: 200 J m^−2^, shear strength: 120 kPa), tunable electrical properties, and long-term structural and electrochemical stability. Electrophysiological studies in rat heart models demonstrate its ability to establish conformal biointerfaces for precise epicardial monitoring and electrical modulation of myocardial infarction. This strategy holds promise for advancing tissue–electronics interfaces, with potential applications in healthcare monitoring, diagnosis, and therapies [49]. Moreover, a fully commercially accessible PEDOT:PSS-based ink is demonstrated for highly 3D-printable fabrication of complex structures, which are converted into high-performance hydrogels via post-printing freeze–thaw treatment. The resulting hydrogels exhibit high conductivity (~2000 S m^−1^), excellent elasticity, water stability, electromagnetic interference shielding, and sensing capabilities. Biocompatibility further highlights their potential for implantable and tissue engineering applications, offering a versatile strategy for customizable multifunctional hydrogel development (Figure 4f) [50].

Printability challenges (e.g., clogging, poor resolution) in extrusion- or ink-based methods (DIW, inkjet) stem from mismatched rheology between PEDOT:PSS and matrix precursors [48]. Key advancements include rheological tailoring (adding nanoclay, cellulose nanofibers, or shear-thinning polymers to achieve shear-thinning behavior for DIW), reactive ink design (microreactive inkjet printing enables in-flight crosslinking to stabilize printed structures without post-processing) and substrate engineering (hydrophobic patterning or sacrificial layers improve resolution and adhesion) [49].

#### 2.2.7. Digital Light Processing

Digital light processing (DLP) uses projected light patterns to photopolymerize hydrogel resins in a layer-wise manner. PEDOTs-functionalized resins enable the rapid prototyping of high-resolution conductive structures (e.g., microneedle arrays) [51]. This method excels in customization but requires expensive equipment and optimized photoactive formulations [52]. DLP printing was used to fabricate GelMA/CS hydrogels integrated with PEDOT nanoparticles through interfacial polymerization, creating conductive pathways. PEDOT incorporation enhanced both electrical conductivity and mechanical properties while preserving printed details. The GelMA/CS-PEDOT hydrogel promoted cell proliferation and axon outgrowth of PC12 and Schwann cells in vitro. Furthermore, direct current electrical stimulation facilitated axon elongation on the conductive substrate. In vivo, a conductive nerve conduit repaired a 10 mm rat sciatic nerve defect, demonstrating the GelMA/CS-PEDOT scaffold’s efficacy in peripheral nerve repair [52].

## 3. Application of PEDOT:PSS-Based Conductive Hydrogels in Wearable Sensors

### 3.1. Mechanical Deformation Monitoring

**Figure 5:**
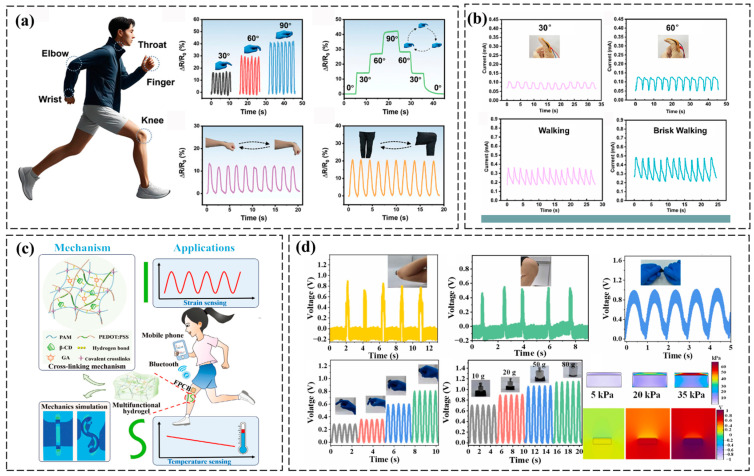
Application of PEDOT:PSS-based conductive hydrogels in mechanical deformation monitoring. (**a**) Monitoring of human motion through resistance changes at five locations: finger bending, finger angles, elbow, wrist, knee joints, and throat during pronunciation [56]. (**b**) The sensor monitors human motions, including finger bending at 30°, 60°, and 90°, as well as walking, brisk walking, and running [57]. (**c**) Schematic diagram of the mechanism and applications of the PEDOT:PSS-based hydrogel [33]. (**d**) The real-time voltage signal output of TAP-PPNM TENG is analyzed across various application scenarios, with the electric potential distribution under different pressure conditions simulated using COMSOL multiphysics software [59]. All pictures have adopted with permission.

PEDOT:PSS-based conductive hydrogels excel in bioelectrical signal monitoring by combining high conductivity for signal fidelity, biocompatibility for seamless tissue integration, and mechanical adaptability to ensure stable, long-term recording of electrophysiological activities such as cardiac, neural, and muscular signals [53,54,55]. For example, super-robust conductive hydrogels with highly oriented, densified networks were developed through stretch-drying-induced assembly, salting-out, and ionic crosslinking. These materials exhibited exceptional mechanical strength (tensile strength ~17.13–142.1 MPa, compared to <1 MPa for most conventional hydrogels) and toughness (50 MJ m^−3^), alongside high conductivity (30.1 S/m) and reliable strain sensing capabilities. The hydrogel demonstrated practical potential in biomimetic electronic skin applications, significantly enhancing signal fidelity and device durability (Figure 5a) [56]. Furthermore, Zhang et al. developed an anti-freezing conductive hydrogel through the integration of polyacrylamide, LiCl, and PEDOT:PSS-coated cellulose nanofibers. The cellulose framework enhanced intermolecular hydrogen bonding, leading to improved mechanical robustness and electrical conductivity. Meanwhile, LiCl promoted strong colloidal–water interactions, enabling flexibility at subzero temperatures. The resulting hydrogels exhibited a high tensile strength of 0.19 MPa, compressive strength of 0.92 MPa, and energy dissipation of 41.9 kJ/m^3^. The optimized hydrogel demonstrated cryogenic stability with maintained structural integrity at extreme low temperatures. This composite material was successfully engineered into flexible sensors capable of precise human motion detection and physiological signal tracking, showcasing potential for advanced wearable technologies (Figure 5b) [57]. The PEDOT:PSS-based hydrogel, synthesized via radical grafting and supramolecular crosslinking, combines high sensitivity (TCR = −1.70% °C^−1^), high toughness (9.31 MJ m^−3^), wide strain range (0–600%), outstanding adhesion strength (36.07 kPa), and multiple biofunctions. Designed as a bimodal sensor, it enables decoupled strain and temperature sensing. An “IS”-shaped wearable system, guided by simulations, integrates the hydrogel with flexible circuits for closed-loop rehabilitation monitoring, advancing self-calibrated, multifunctional wearable devices (Figure 5c) [33].

Self-powered hydrogels enable flexible, energy-autonomous sensors for real-time human joint motion tracking in wearable healthcare systems [58]. For instance, a PEDOT molecular bridging strategy enables robust interfacial integration between hydrogel electrodes and adjacent sensing layers. A polyacrylamide-PEDOT dual-network hydrogel (PPNM) electrode was fabricated via dual crosslinking, while vacuum-assisted dip-coating sequentially deposits silver nanowires and PPNM onto TPU foam, forming a 3D dual-conductive network (TAP). Interfacial PEDOT bridges between TAP and PPNM ensured stable electron transport under extreme mechanical deformation, overcoming interfacial mismatch in stretchable sensors (Figure 5d) [59]. Moreover, Sun et al. fabricated an all-polymer conductive microfiber (PU@PVA-PEDOT:PSS SI-CF) via microfluidic spinning technology, integrating a strain-insensitive semi-interpenetrating hydrogel core into a helical architecture. By optimizing MST parameters, the microfiber achieves exceptional stretchability (up to 500%), high conductivity (147 S cm^−1^), strain-insensitive conductance (<5% resistance fluctuation at 100% strain), and cyclic durability (2000 stretching-releasing cycles). Its decoupled mechanical-electrical properties enable multifunctional applications in stretchable interconnects, self-powered sensors, and flexible electrothermal devices for smart wearables [40].

### 3.2. Tissue Microenvironment Monitoring

**Figure 6:**
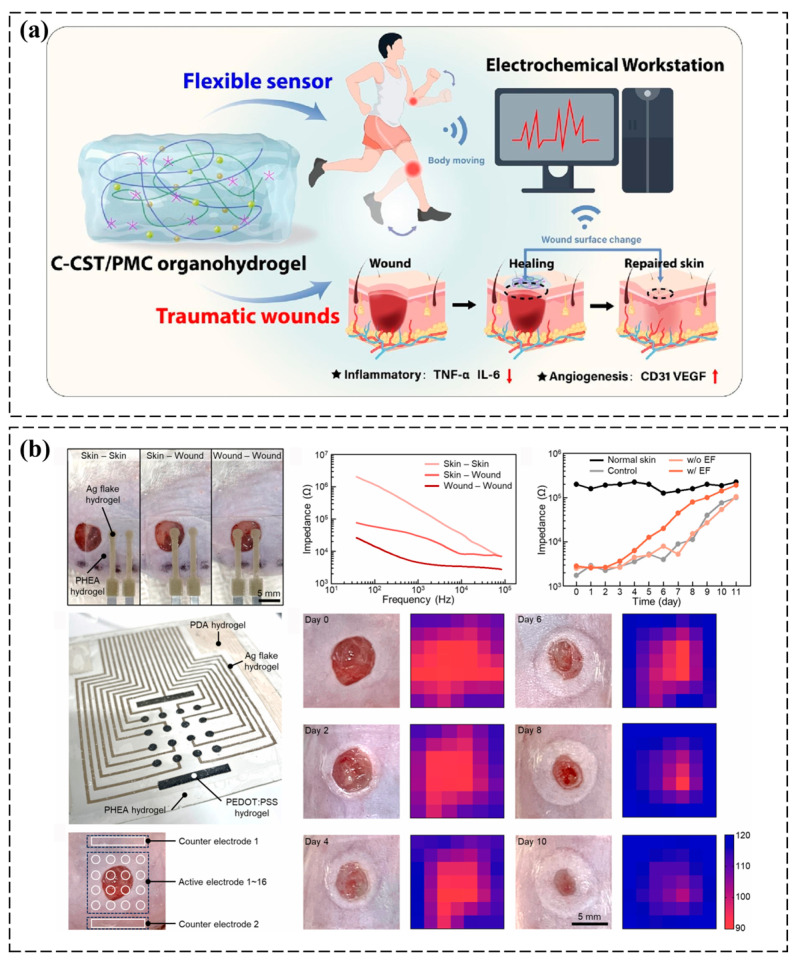
Application of PEDOT:PSS-based conductive hydrogels in tissue microenvironment monitoring. (**a**) A multifunctional conductive C-CST/PMC organohydrogel with excellent mechanical properties, self-healing, antibacterial, and biocompatibility was synthesized, showing promising wound-healing and real-time monitoring potential as a wearable sensor in diabetic rats and medical applications [62]. (**b**) Impedance monitoring and 2D mapping were used to track the wound healing process in a mouse model, utilizing PEDOT:PSS hydrogel electrodes to measure impedance at various skin sites and visualize the healing through frequency-dependent impedance and 2D mapping during EF stimulation and iontophoretic drug delivery [63]. All pictures have adopted with permission.

PEDOT:PSS hydrogels facilitate the real-time monitoring of biochemical fluctuations in tissue microenvironments through their ion–electron coupled conductive networks, advancing precision diagnostics for chronic wounds [60,61]. A multifunctional conductive organohydrogel (C-CST/PMC) was engineered through the synergistic integration of catechol-modified chitosan, silk nanofibrils, and tannic acid within a PVA-based binary solvent system. This material demonstrates exceptional mechanical robustness, cryogenic flexibility (−18 °C)—the ability to maintain pliability and resist brittleness at subzero temperatures due to (1) a glycerol–water binary solvent that suppresses ice crystallization and (2) dynamic hydrogen/coordination bonds that enable energy dissipation under thermal stress—autonomous self-healing, and dual antibacterial–antioxidant functionality, while maintaining high ionic conductivity across extreme temperatures [60,61]. Its bioactive interface promotes angiogenesis and suppresses inflammation in diabetic wounds, as validated by accelerated in vivo healing. Furthermore, when integrated into bionic wearable sensors, the hydrogel enables the real-time monitoring of wound recovery dynamics, establishing a closed-loop platform for simultaneous therapeutic intervention and physiological tracking in skin trauma management (Figure 6a) [62]. Additionally, Shin et al. developed an all-hydrogel electronic skin (e-skin) patch using photolithography-compatible functional hydrogels, including PEDOT:PSS-based electrodes with tissue-mimetic hydration and mechanical compliance. The hydrogel’s human-tissue-like modulus and water content ensured stable biointerfacing in dynamic physiological conditions. In murine models, the e-skin synergistically accelerated wound healing through electric field-enhanced fibroblast activity and iontophoretic drug delivery, while simultaneously mapping healing progress via impedance changes. This dual-action platform, integrating therapeutic stimulation with real-time biosensing, established a paradigm for closed-loop tissue-interfacing systems in clinical diagnostics and regenerative medicine (Figure 6b) [63].

### 3.3. Electrophysiological Monitoring

**Figure 7:**
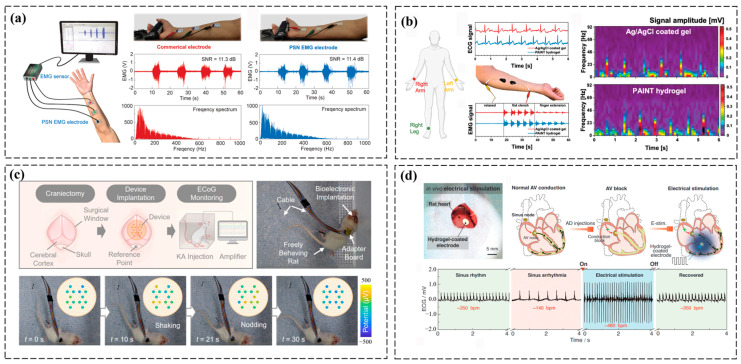
Application of PEDOT:PSS-based conductive hydrogels in electrophysiological monitoring. (**a**) A schematic illustration of the fabricated skin electrodes for EMG signal recording shows a comparison of the recorded EMG signals and frequency spectrum between our PSN hydrogel-based electrodes and commercial electrodes [66]. (**b**) A schematic of the electrode setup for ECG and sEMG tests, with signals recorded using Ag/AgCl gel and PAINT hydrogel electrodes, and the ECG frequency-amplitude spectrogram [67]. (**c**) Epilepsy monitoring in freely moving rats, including key experimental steps, in vivo ECoG recording, and observed epileptic behaviors such as shaking and nodding [69]. (**d**) In vivo treatment of AV block using a conducting polymer hydrogel-coated bioelectrode, restoring heart rhythm through ventricular epicardial pacing [70]. All pictures have adopted with permission.

PEDOT:PSS-based conductive hydrogels excel in bioelectrical signal monitoring by combining high conductivity for signal fidelity, biocompatibility for seamless tissue integration, and mechanical adaptability to ensure stable, long-term recording of electrophysiological activities such as cardiac, neural, and muscular signals [64,65]. For example, Yu et al. engineered a PEDOT:PSS hydrogel with exceptional conductivity, intrinsic softness, and mechanical robustness through PSS-chain engineering using thermally crosslinkable N-(hydroxymethyl)acrylamide. This hydrogel achieves unprecedented electromechanical synergy, combining tissue-like compliance, robust elasticity, and high energy dissipation. The material’s processability is further harnessed by formulating a shear-thinning ink for multi-material 3D printing, enabling the direct fabrication of skin-conformal electrodes with performance parity to clinical-grade soft bioelectronics (Figure 7a) [66]. Moreover, a bioinspired conductive hydrogel was engineered via physicochemical crosslinking of biopolymers for the direct ink writing of epidermal electrodes. This supramolecular design enabled rapid gelation and submillimeter-scale resolution using handheld extrusion systems. Compared to conventional Ag/AgCl gel electrodes, the in situ-formed hydrogel demonstrates enhanced electromyographic signal fidelity and reduced stimulation current demand during facial neuromuscular interfacing, attributed to its adaptive contact impedance via skin-conformal sol–gel transition. The mechanoelectronic coupling strategy pioneered on-demand fabrication of medical-grade wearable electronics with improved biosignal acquisition and therapeutic precision (Figure 7b) [67].

Furthermore, PEDOT:PSS-based conductive hydrogels serve as dual-functional biosensing interfaces, enabling non-invasive surface electromyography and implantable monitoring of intramuscular/cardiac bioelectrical activities [38,68]. For instance, Lao et al. engineered a catechol-functionalized PEDOT hydrogel with tissue-mimetic mechanical compliance and universal adhesion, leveraging nanoscale PEDOT percolation networks to achieve strain-insensitive conductivity. This dynamic catechol-mediated bioadhesion enables seamless bioelectronic-tissue integration, sustaining high-fidelity bioelectrical recordings (EMG/ECG/ECoG) under physiological motion through optimized interfacial impedance matching. Its capacity for motion artifact-resistant neural interfaces advances minimally invasive diagnostics and closed-loop neuromodulation systems (Figure 7c) [69]. Xue et al. developed a universal nanoscale interface engineering strategy to construct fatigue-immune conductive hydrogel coatings on metallic bioelectrodes through nanocrystalline domain regulation at hydrogel–substrate interfaces. This biomimetic coating achieves simultaneous electrochemical stability and mechanical compliance, reducing cardiac pacing thresholds while ensuring chronic stimulation stability through optimized charge injection efficiency. The fatigue-immune mechanism, arising from energy-dissipative nanocrystalline networks, enables seamless integration with dynamic cardiac tissues, demonstrating transformative potential for high-performance bioelectronic implants in cardiac rhythm management (Figure 7d) [70].

## 4. Conclusions and Future Perspectives

PEDOT:PSS-based conductive hydrogels have emerged as promising materials for wearable sensors due to their excellent conductivity, mechanical adaptability, and biocompatibility. This review highlights key gelation strategies, including polymer crosslinking and ionic interactions, along with advanced fabrication techniques such as electrospinning and inkjet printing, which enhance their performance in health monitoring applications. These hydrogels enable the real-time detection of mechanical deformations, tissue microenvironments, and electrophysiological signals, making them valuable for continuous health monitoring.

The rapid evolution of PEDOT:PSS-based conductive hydrogels heralds a transformative era in health monitoring, yet several frontiers demand exploration to unlock their full potential. First, multi-material convergence, integrating stimuli-responsive polymers, 2D nanomaterials, and bioactive components, could yield hydrogels with self-diagnostic and on-demand therapeutic functions, enabling closed-loop systems for chronic disease management. Second, advances in 4D printing and AI-driven microstructure optimization may overcome current limitations in manufacturing scalability and resolution, facilitating patient-specific sensor architectures that adapt dynamically to anatomical changes. Third, the development of energy-autonomous systems through triboelectric or enzymatic energy harvesting could eliminate external power dependencies, critical for implantable or long-term wearable applications. Addressing long-term biofouling resistance and mechanical degradation under extreme conditions (e.g., sweat corrosion, cyclic loading) remains imperative, necessitating covalent/noncovalent dual-network engineering and antifouling surface topographies.

Moreover, it is crucial to contextualize hydrogel-based platforms within the broader field of organic bioelectronics. Emerging modalities such as organic electrochemical transistors (OECTs) and ionic cable-based interfaces offer alternative architectures for bio-signal acquisition and transmission [71,72]. OECTs, with their high transconductance and ion-sensitive gating mechanisms, provide superior signal amplification and are particularly suited for soft, hydrated environments similar to those where conductive hydrogels operate [73,74]. Ionic cables, by contrast, enable long-range signal propagation through ionic currents, which is advantageous for distributed sensing networks in soft electronics [75]. While PEDOT:PSS hydrogels excel in conformability, mechanical tunability, and intimate tissue integration, they may face challenges in complex circuit integration and signal fidelity compared to OECTs. Therefore, exploring hybrid systems that synergistically combine hydrogel matrices with transistor-based or ionic communication components may pave the way for multifunctional, next-generation bioelectronic interfaces.

Furthermore, establishing standardized protocols for clinical validation and regulatory compliance will accelerate translation from lab-scale prototypes to FDA-approved medical devices. By synergizing materials innovation with digital health technologies, PEDOT:PSS hydrogels are poised to redefine personalized healthcare through centimeter-scale epidermal arrays for whole-body physiology mapping and minimally invasive neural interfaces for brain–machine integration.

## Figures and Tables

**Table 1:** The relative advantages and disadvantages of physical, chemical, and hybrid crosslinking methods.

Criteria	Physical Crosslinking	Chemical Crosslinking	Hybrid Crosslinking
Conductivity	High (Dynamic ionic networks enable free ion mobility)	Moderate (Rigid networks may restrict ionic transport)	High (Combined ionic/covalent pathways synergize charge transfer)
Mechanical Strength	Low-Moderate (Reversible bonds limit stability)	High (Irreversible covalent bonds create robust networks)	Tunable (Balanced covalent rigidity and dynamic adaptability)
Biocompatibility	Excellent (No toxic residues; natural polymer compatibility)	Limited (Potential cytotoxicity from residual crosslinkers)	Moderate (Depends on crosslinker type; reversible interactions reduce toxicity risks)
Scalability	High (Simple processing; ambient conditions)	Moderate (Requires precise chemical control; post-treatment needed)	Moderate-High (Adaptable to multiple fabrication methods)
Key Advantages	Dynamic self-healing Biodegradable Mild preparation	Structural stability High tensile strength Long-term durability	Programmable mechanics Stimuli-responsiveness Synergistic performance
Key Limitations	Weak mechanical resilience Environmental sensitivity	Limited flexibility Biocompatibility concerns	Complex optimization Potential phase separation
Typical Applications	Wearable sensors, transient bioelectronics	Structural scaffolds, implantable devices	Multifunctional sensors, adaptive soft robotics

## Data Availability

No new data were created or analyzed in this study. Data sharing is not applicable to this article.
